# Thermal Infrared Imagery Integrated with Terrestrial Laser Scanning and Particle Tracking Velocimetry for Characterization of Landslide Model Failure

**DOI:** 10.3390/s20010219

**Published:** 2019-12-30

**Authors:** Junwei Ma, Xiaoxu Niu, Xiao Liu, Yankun Wang, Tao Wen, Junrong Zhang

**Affiliations:** 1 Three Gorges Research Center for Geo-Hazards of the Ministry of Education, China University of Geosciences, Wuhan 430074, China; majw@cug.edu.cn (J.M.); niuxx@cug.edu.cn (X.N.); 2 Faculty of Engineering, China University of Geosciences, Wuhan 430074, China; yankun_wang@cug.edu.cn (Y.W.); zjr@cug.edu.cn (J.Z.); 3 School of Geosciences, Yangtze University, Wuhan 430100, China; wentao200840@yangtzeu.edu.cn

**Keywords:** landslide failure, thermal infrared imagery, terrestrial laser scanning, particle tracking velocimetry

## Abstract

A laboratory model test is an effective method for studying landslide risk mitigation. In this study, thermal infrared (TIR) imagery, a modern no-contact technique, was introduced and integrated with terrestrial laser scanning (TLS) and particle tracking velocimetry (PTV) to characterize the failure of a landslide model. The characteristics of the failure initiation, motion, and region of interest, including landslide volume, deformation, velocity, surface temperature changes, and anomalies, were detected using the integrated monitoring system. The laboratory test results indicate that the integrated monitoring system is expected to be useful for characterizing the failure of landslide models. The preliminary results of this study suggest that a change in the relative TIR signal (ΔTIR) can be a useful index for landslide detection, and a decrease in the average value of the temperature change ($\bar{\Delta TIR}$) can be selected as a precursor to landslide failure.

## 1. Introduction

Landslide movements and failures cause severe economic losses and human deaths worldwide. According to statistics, 55,997 people were killed by 4862 fatal landslides from January 2004 to December 2016 [1]. Recently, a fatal landslide occurred on the evening of 22 July 2019 in Shuicheng County, Liupanshui city, Guizhou, China. This event resulted in 32 missing people and the deaths of 13 people.

Field monitoring and laboratory model tests have been demonstrated to be useful methods for monitoring and studying landslide risk mitigation. With the rapid development of modern monitoring instruments and techniques, significant advances have been achieved in the field of landslide monitoring over the past ten years. Terrestrial laser scanning (TLS), a modern geodetic technique, is capable of providing huge amounts of high-resolution, high-accuracy data on the 3D coordinates of target object over a very short time. With the advantages of fast and noncontact data acquisition, the TLS technique is suitable for collecting point clouds for landslide monitoring, especially for high steep landslides with limited access. This technique has been widely applied in landslide deformation monitoring in the field [2,3]. Additionally, synthetic aperture radar interferometry (InSAR) is a powerful remote sensing technique with a high degree of accuracy and wide spatial and temporal coverage and has become an increasingly popular approach for landslide monitoring in the field [4,5]. Recently, the use of unmanned aerial vehicles (UAVs), a modern remote sensing technique with high spatial resolution, has been successfully applied for landslide monitoring in the field [6,7,8]. The key advantage of the UAV technique is that it is capable of providing images of areas that are difficult or impossible to access at low cost. Furthermore, the UAV technique bridges the gap between terrestrial observations and satellite sensor observations in terms of scale and resolution. Other advanced techniques, including thermal infrared (TIR) imagery and distributed fiber optic sensing, have been successfully applied for landslide monitoring in the field [9,10,11].

TIR imagery is a modern noncontact technique measuring the radiation pattern of a target object and converting it into a thermal image [12]. It has been successfully applied to manage hazards, such as fires, coal mine fires, and volcanic activity [13,14]. However, application of TIR imagery in the study of landslides is very limited. The first pioneering attempts to detect unstable landslide areas using thermal maps were conducted by Japanese scientists in the early 1990s [15,16]. However, there was little further investigation until the late 2010s [9,10]. Recently, the application of thermal infrared imagery in landslide studies has generally been performed over limited time spans. Moreover, the application of TIR imagery to characterize landslide failure is rarely reported.

A laboratory model test is effective for studying landslide risk mitigation. Due to size limitations, the monitoring of laboratory model tests lags behind related fields of research [17]. The existing monitoring instruments for laboratory tests, e.g., laser displacement sensors, earth pressure sensors, moisture content sensors, and pore-water pressure sensors [18,19,20,21], are traditional instruments widely used in field-based monitoring but are small in size. Recently, some novel instruments and techniques have been designed and tested for monitoring landslide laboratory tests. A flexible inclinometer probe was designed for monitoring deep displacement in laboratory tests [17]. Aiming to monitor the sliding surface, a novel monitoring instrument integrating fiber Bragg grating and a polyvinyl chloride tube was designed by Wang et al. and tested in a laboratory test [22]. The feasibility of monitoring using the elastic wave velocity technique has been explored by Chen et al. [23]. A new piezoelectric sensor employing self-structure pressure distribution and capacitive circuit voltage distribution methods has been presented by Li et al. for monitoring dynamic force in a laboratory model test [24].

To enhance the ability to monitor laboratory tests and to investigate the characteristics of landslide failure, a novel monitoring system integrating TIR imagery, TLS, and PTV is presented for the characterization of the failure of a landslide model.

## 2. Materials and Methods

### 2.1. Descriptions of the Landslide Model Test

Movement and failure of landslide can be induced by many different causes [25], including rainfall and thrust loading. Due to rapid urbanization, construction of buildings, spoil, vehicle dynamic loads, or surcharge loads will increase the driving forces resulting in thrust load-caused landslide [26]. In the present study, a model test was performed to investigating the characteristics of failure caused by thrust loading.

The landslide model test conducted in this study was performed in a rigid steel box. The landslide model is mainly composed of a landslide body and sliding zone. The landslide body is 1.76 m long and 0.9 m wide. The average thickness of the landslide body is 0.25 m. The average inclination of the landslide surface and sliding zone are 22° and 19°, respectively. More details about the landslide model are available in Table 1.

**Figure 1:**
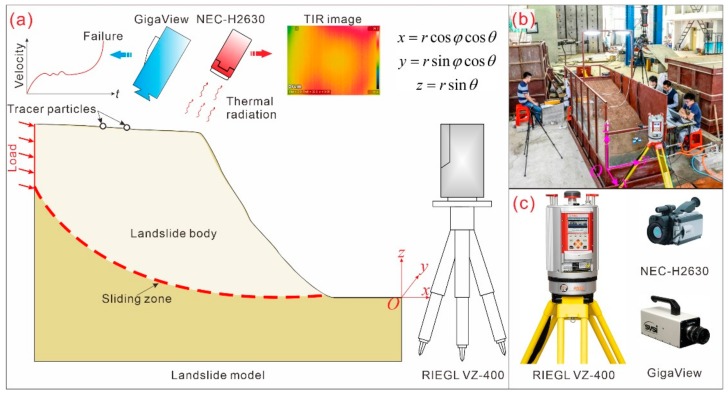
(**a**) General arrangement of the landslide model and integrated monitoring system. (**b**) Photograph of the landslide model test and integrated monitoring system. (**c**) Photograph of each component of the monitoring system.

Multiple-stage external loads were applied at the head of the model to simulate the landslide movement and failure (schematically illustrated in Figure 1). Each stage consists of 20 min of ramp loading and 40 min of holding. The model test lasted 8.52 h. At the beginning of the test, only small deformations at centimeter magnitude were detected and no surface cracks were observed. As the loading increased to 1368 N, a crack perpendicular to the sliding direction formed near the head of the slope. Rapid failure characterized by a significant decrease in the magnitude of the load was observed at the peak load of 1873.32 N. A sliding surface was formed within the sliding zone soil.

### 2.2. Integrated Monitoring System

An integrated monitoring system comprising TIR imagery (NEC-H2630, Nippon Avionics Co., Ltd., Tokyo, Japan), TLS (RIEGL VZ-400, RIEGL Laser Measurement Systems GmbH, Horn, Austria), and PTV (GigaView, Southern Vision Systems Inc., Madison, WI, USA) is presented in this study for monitoring and characterizing the failure of a landslide model (schematically illustrated in Figure 1).

#### 2.2.1. TIR Imagery

**Figure 2:**
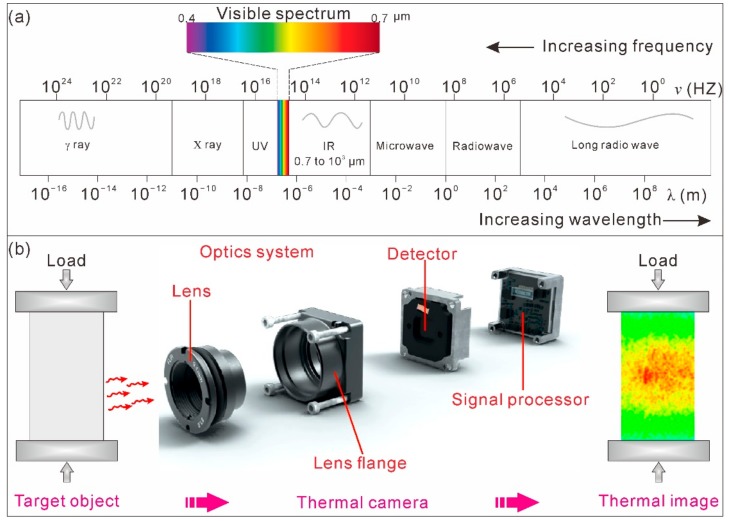
(**a**) Electromagnetic spectrum and infrared (IR) band. (**b**) The basic working principle of a thermographic camera.

The basic principle of TIR imagery is that any object with a temperature above absolute zero absorbs or emits thermal energy in the infrared (IR) range [9,10]. TIR imagery focuses and detects the infrared energy emitted by objects and converts the data into a color scale image with a special color palette (schematically illustrated in Figure 2).

In the electromagnetic spectrum shown in Figure 1a, the IR band is located between visible light and microwaves, extending from approximately 430 MHz (with a wavelength of approximately 0.7 μm) to 300 GHz (with a wavelength of approximately 10^3^ μm). The IR band can be further subdivided into the following subbands: near IR (with wavelengths of 0.7 to 1 μm), short wave IR (with wavelengths of 1 to 3 μm), mid wave IR (with wavelengths of 3 to 5 μm), and long wave IR (with wavelengths of 8 to 14 μm). The wavelengths of IR are longer than those of visible light. Therefore, IR is generally invisible to human eyes but can be captured by an infrared thermographic camera. An infrared thermographic camera is typically composed of an optics system, detector, and signal processor (Figure 2b).

In a thermal image with a color scale, the temperature of each pixel is based on the integrated infrared radiance over the covered area. Different temperature values are mapped as different colors, thus allowing a visual representation of the heat profile of the target object. Usually, brighter colors, e.g., red, orange, and yellow, represent warmer temperatures, which indicate the emission of more heat and infrared radiance. Darker colors, e.g., purples and dark blue, represent cooler temperatures, which indicate the emission of less heat and infrared radiance.

In this study, a NEC-H2630 thermographic camera (Nippon Avionics Co., Ltd., Tokyo, Japan) with a measuring range of −40 to 500 °C and a resolution of 0.04 °C was placed 1.5 m above the model surface to measure the TIR during the model test. The thermal thermographic camera was positioned with a perpendicular orientation. More specific details of the NEC-H2630 thermographic camera are available in Table 2. Several measures, including performing the test and monitoring on a cloudy day, restricting access to the monitoring area and closing windows and curtains, were implemented to further eliminate environmental radiation effects.

#### 2.2.2. TLS

In the present study, a cutting-edge RIEGL VZ-400 TLS (RIEGL Laser Measurement Systems GmbH, Horn, Austria) utilizing time-of-flight method (TOF) technology was adopted for deformation monitoring of the landslide model. This RIEGL VZ-400 TLS provides point cloud data with 5 mm accuracy and 3 mm repeatability at very high speeds (up to 122,000 measurements/second) with the measurement range from 1.5 to 600 m. More features of the RIEGL VZ-400 TLS are listed in Table 2.

#### 2.2.3. PTV

PTV is a nonintrusive image-based approach used to trace the velocity of individual particles along trajectories and has been widely applied to determine the particle velocities in both laboratory and field settings [27,28,29,30,31,32,33]. It mainly consists of two steps: individual particle identification and particle tracking. In the process of individual particle identification, recorded images are enhanced for viewing through filtering and thresholding, and the particle centroids in the frame are restored. In the process of particle tracking, the sequence of the particle centroid is identified from consecutive image frames to determine the particle velocity. In this study, a GigaView high-speed digital camera (Southern Vision Systems Inc., Madison, WI, USA) was adopted. This camera allows a maximum of 17,000 frames to be captured per second. The specifications of the GigaView camera are listed in Table 2. In this study, 224 white dots with a diameter of 8.5 mm were placed on the model surface as tracer particles. Tracer particles were also set as reference points to manually align the observations from TLS and TIR. The GigaView high-speed camera was placed at an elevation of 1 m above the model surface to record the motion at a temporal resolution of 30 frames and a spatial resolution of 1280 by 1024 pixels. The recording of a high-speed camera was synchronized with a light-emitting diode (LED).

### 2.3. Data Preprocessing

**Figure 3:**
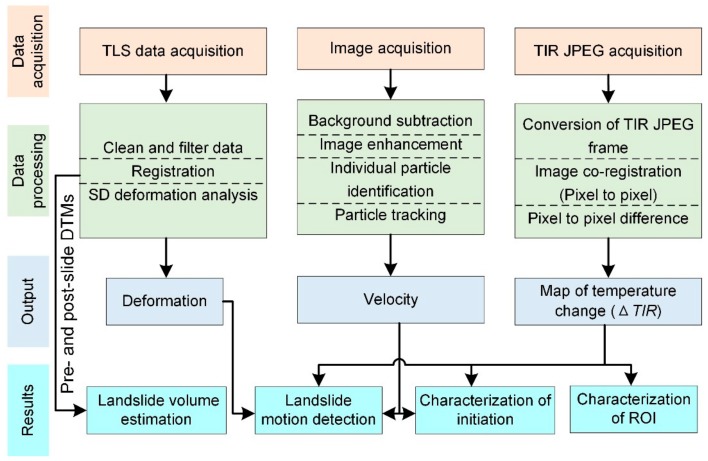
Flow chart showing the data processing for characterization of landslide failure using the integrated monitoring system.

The overall framework of data processing for the characterization of landslide failure using the integrated monitoring system is shown in Figure 3. Prior to loading, the landslide model was scanned and recorded by the TLS system and the TIR thermographic camera as a reference. During the model test, the TIR thermographic camera and GigaView high-speed camera were continuously recording, while TLS system performed scans every five minutes. In each scan, a point cloud of approximately 2 million points was obtained.

The TIR JPEGs acquired from the NEC-H2630 thermographic camera were processed through conversion of the TIR JPEG frame, image coregistration, and pixel-to-pixel difference. First, the TIR raw files in JPEG format were converted into CSV files throughFLIR R&D software version 3.4. Due to the model movement, the area of the TIR frame varies over time. A coregistration was performed using the free MATLAB algorithm SIFT flow [34]. SIFT flow is a flow-based image aligning algorithm that matches pixel-to-pixel correspondences between two images. After the aligning procedure, a pixel-to-pixel comparison was performed to determine the changes in temperature (ΔTIR). To compare thermal characteristics between the deformation area and the stable area, maps of temperature change were registered with the TLS data by manually picking two reference tracer particles.

The point clouds obtained from TLS were processed with data cleaning and filtering processes, registration, and shortest distances (SDs) deformation analysis. The point clouds were registered to a local coordinate system located at the corner of the rigid steel box (see Figure 1 for local coordinate system information). The surface deformation was obtained through SDs point-to-point comparison algorithms. The SDs comparison algorithm allows the detection of vertical, horizontal, and oblique distances. In the present study, positive deformation indicates that a point in the test point cloud is in front of that in the reference point cloud. Negative deformation indicates that a point in the test point cloud is behind that in the reference point cloud, resulting from vertical settlement or subsidence. The cleaned and filtered point clouds were also meshed to generate high-resolution digital elevation models (DEMs). The landslide volume was estimated by computing the differences between pre- and post-slide DEMs.

The PTV analysis was carried out using MicroVec V3 (Microvec Pte. Ltd. Gambas, Singapore) to determine the velocity of individual particles on the model surface.

## 3. Results

### 3.1. Characteristics of Landslide Motion and Initiation

#### 3.1.1. Landslide Volume Estimation

Pre- and post-slide DEMs were generated from the obtained TLS point clouds. The landslide volume was estimated from a comparison of the pre- and post-slide DEMs. The DEM comparison indicates that the sliding volume of the landslide model is equal to 0.218 m^3^.

#### 3.1.2. Landslide Initiation and Motion

**Figure 4:**
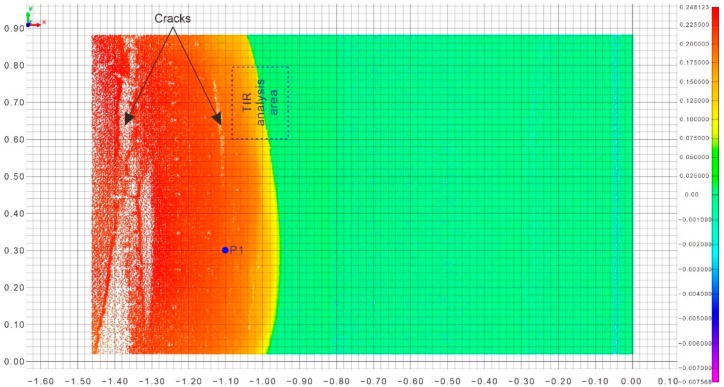
Shortest distance (SD) deformation distribution after the failure of the landslide model obtained from point cloud comparison.

SD displacement of the post-slide model obtained through point cloud comparison is shown in Figure 4. When the failure of the landslide model occurred, the maximum displacement was 0.25 m. Positive displacements in warm color indicate that the model surface moved forward and that the test point cloud is in front of the reference point cloud. The displacement magnitude deceased from the upslope region to the downslope region. In Figure 4, the deformation in the downslope region is represented by cold colors, which indicates that no significant movements occurred and that these areas were stable. The above results indicate that the deformation occurred first in the uphill region and progressed downslope. Two noticeable cracks perpendicular to the sliding direction formed in the upslope region. The major crack was approximately 5 cm wide and developed into a continuous crack along the model surface.

**Figure 5:**
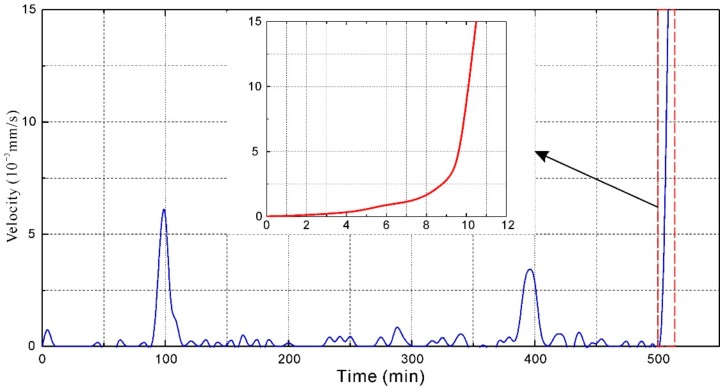
Velocity of tracer particle P1 obtained from particle tracking velocimetry (PTV) (location of the particle is shown in Figure 4).

A typical tracer particle located in the upslope region named P1 was selected for PTV analysis. The velocity of P1 obtained from PTV analysis is shown in Figure 5. The obtained velocity fluctuated in the test. Several rapid, short-duration movements with significant velocity occurred. When the model failure occurred, the velocity monitoring curve shows an exponential growth trend. The displacement increased substantially to 15 × 10^−3^ mm/s.

**Figure 6:**
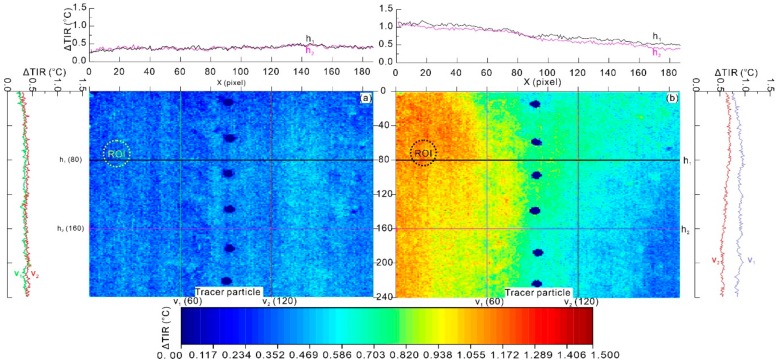
Spatial distribution of the change in the relative thermal infrared signal (ΔTIR) by pixel-to-pixel comparison. (**a**) In the middle of the model test (t = 4.25 h), (**b**) before the landslide model failure (t = 8.42 h) (location of the ΔTIR region is shown in Figure 4).

A small area with a spatial resolution of 187 × 239 pixels covering the landslide area and the nonlandslide area was extracted to show the thermal characteristics of these areas during the model test. The ΔTIR during the model test was mapped to a blue-to-red rainbow color gradient and shown in Figure 6. Two horizontal and two vertical profiles were selected to show surface temperature changes parallel to and perpendicular to the sliding direction. The ΔTIR data show that in the middle of the model test (t = 4.25 h), the surface temperature ((a) in Figure 6) increased slightly, with an average increase of 0.382 °C, due to energy accumulation by loading. The ΔTIR data in the parallel profiles were approximately equivalent. There was no significant difference between the deformation area and the stable area.

Before the occurrence of the model failure (t = 8.42 h, ((b) in Figure 6)), the average value of ΔTIR increased to 0.751 °C. A distinct ΔTIR difference was observed between the deformation area and the stable area. The ΔTIR in the landslide deformation area was significantly higher than that in the stable area. A ΔTIR decrease trend can be observed along the two selected horizontal profiles. These ΔTIR differences were induced by energy accumulation caused by elastic–plastic deformation, surface energy, friction and heat in the deformation area. Moreover, those findings correspond well with previous field observations [15,16] indicating the surface temperature in landslide deformation area is higher than that in non-landslide areas. This finding shows that ΔTIR can be a useful indicator for differentiating landslide deformation areas from stable areas.

### 3.2. Characteristics of ROI

A region of interest (ROI) with a circular shape was selected from the ΔTIR image for further analysis of the thermal characteristics during the model test. The average value of temperature changes ($\bar{\Delta TIR}$) for the ROI and the room temperature are listed in Table 3. As shown in Table 3, the room temperature was approximately constant during the test. This result indicates that the measures eliminating environmental radiation effects were effective. For the selected ROI, $\bar{\Delta TIR}$ increased with energy accumulation by loading but decreased slightly before model failure occurred. The $\bar{\Delta TIR}$ value of the ROI was very small, with a value close to zero at the beginning of the test (0 to 2.3 h). Then, a period with an increasing trend was observed from 2.8 h to 7.0 h during the test. However, a short period with a slight decrease was observed from 7.3 h to 8.7 h. This decrease was caused by energy dissipation. This finding indicates that a decrease in $\bar{\Delta TIR}$ can represent a precursor to landslide failure.

## 4. Discussion

With the aim of advancing the monitoring of laboratory model tests, TIR imagery has been used to monitor and investigate the thermal characteristics of the failure of a landslide model. The experimental results show that with some proper measures in a controlled environment, TIR imagery is capable of characterizing landslide failure with satisfactory performance.

However, the acquisition and processing of thermal images is a difficult task. Several aspects, including monitoring distance and orientation, material emissivity, weather conditions, and test environment, must be considered to obtain thermal images with satisfactory resolution and accuracy.

First, monitoring distance and orientation have a crucial influence on the measurement accuracy. Generally, due to increases in the integrated effect and atmospheric attenuation effect, the accuracy decreases with increasing viewing distance between the monitored object and the infrared thermographic camera.

**Figure 7:**
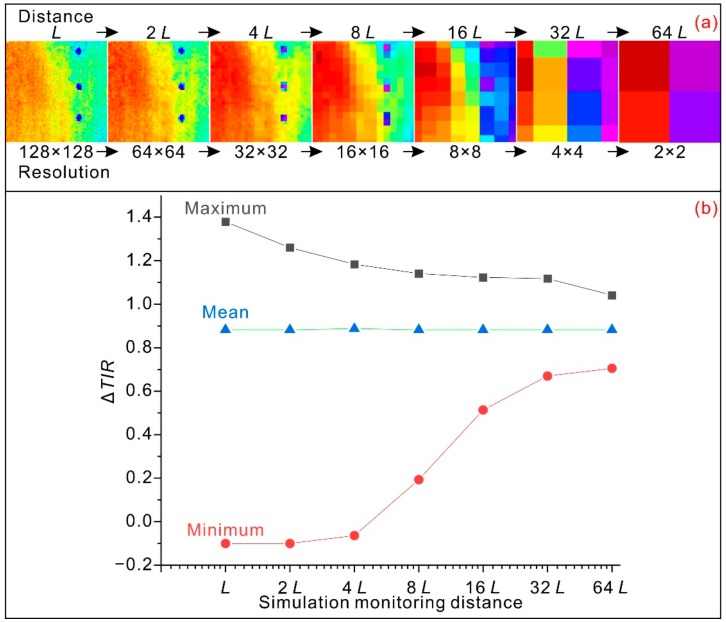
(**a**) Graph showing decreasing thermal image resolution with increasing monitoring distance. (**b**) Graph showing the trends of the minimum, maximum, and mean ΔTIR values in simulated images with increasing monitoring distance.

A simulation test was performed in this study to provide further insight into the effect of monitoring distance on measured temperatures. The atmospheric attenuation effect was ignored in the simulation test. The increase in viewing distance was simulated by a reduction in the resolution of the obtained thermal image. For a fixed monitoring area, when the viewing distance increases from the original distance L to 2^n^ L, the thermal image resolution decreases to 0.5^n^ × 0.5^n^ of the original resolution. A 128 × 128-pixel thermal image extracted from Figure 6b was used in the simulation test. The simulation test mainly consists of the following steps: averaging the infrared radiances over the new pixel area (from 64 × 64 to 2 × 2) and converting the new radiance value into a temperature value. Maximum and minimum mean values were computed from the newly obtained thermal image. The obtained results are shown in Figure 7. The obtained thermal images show that at a greater viewing distance, some important characteristics of the target will be missed in the thermal image. As the viewing distance increases, the maximum ΔTIR decreases towards the mean value due to the integration of infrared radiance over larger covered areas. However, the minimum value shows an increasing trend.

From the above analysis, we recommend that for indoor practical applications, a short monitoring distance close to zero would provide a thermal image with satisfactory resolution and accuracy. When an infrared thermographic camera is placed at a close distance, such as a few meters, the integrated averaging effect of the pixel covered area is reduced. Moreover, the atmospheric attenuation effect is also reduced.

Monitoring orientation mainly influences the amount of emitted thermal energy captured by the infrared thermographic camera. The perpendicular orientation is preferred to capture more thermal radiation energy.

Additionally, the emissivity difference of heterogeneous materials will introduce bias into TIR imagery monitoring. It is highly recommended to compensate for those biases by taking into consideration the thermal gradient of the material.

Furthermore, weather conditions also have a significant influence on the data acquisition. Generally, the emitted thermal energy will be easily detected and captured by an infrared thermographic camera if there are high surface temperature differences between the target object and the surrounding environment. Poor weather conditions, such as strong wind and rain, have a negative influence on the data acquisition. Air convection caused by strong wind and evaporation and subsequent cooling caused by rain will introduce significant bias into outdoor thermal data acquisition. Therefore, the feasibility of using TIR imagery for rainfall-induced landslide model test still holds. Cloudy days are preferred for satisfactory data acquisition.

Considering the abovementioned factors, the following measures are recommended for indoor tests: performing the test on a cloudy day, restricting access to the monitoring area and closing windows and curtains. Analysis of temperature changes and comparison between the target object and a nearby zone are recommended to mitigate bias induced by the reflected apparent temperature and to minimize background or atmospheric interferences.

Although very promising, TIR imagery alone is insufficient for a complete characterization of landslide failure. To obtain a comprehensive analysis, the valuable TIR imaging technique should be integrated with other techniques, such as TLS and PTV.

## 5. Conclusions

In this study, TIR imagery, a noncontact technique, is presented and integrated with TLS and PTV to characterize the failure of a landslide model. The initiation, motion, and ROI characteristics, including landslide volume, deformation, velocity, surface temperature change, and anomalies, were detected using the integrated monitoring system. A relative TIR indicator (ΔTIR) representing changes in temperature was chosen to analyze the thermal characteristics and to identify anomalies associated with landslide failure. The experimental results show that the integrated monitoring system is promising for the characterization of landslide failure. The ΔTIR in the landslide deformation area was significantly higher than that in the stable area. A short period with a slight decrease in the average value of the temperature change ($\bar{\Delta TIR}$) was observed before the failure of the landslide model. ΔTIR can be a useful indicator for differentiating landslide deformation areas from stable areas. A decrease in $\bar{\Delta TIR}$ can be selected as a precursor of landslide failure.

## Figures and Tables

**Table 1:** Main features of the landslide model test.

Term	Value	Term	Value
Length of the landslide body (m)	1.76	Cohesion of the landslide body (kPa)	3.9
Width of the landslide body (m)	0.9	Cohesion of the sliding zone (kPa)	5.6
Average thickness of the landslide body	0.25	Friction angle of the landslide body (°)	26.8
Average inclination of the surface (°)	22	Friction angle of the sliding zone (°)	18.1
Average inclination of the sliding zone (°)	19	Elastic modulus of the landslide body (MPa)	2.5
Volume of the landslide body (m^3^)	0.405	Elastic modulus of the sliding zone (MPa)	2.2
Density of the landslide body (kg/m^3^)	22.1	Duration (h)	8.52
Density of the sliding zone (kg/m^3^)	17.1	Peak load (N)	1873.32

**Table 2:** Main features of the NEC-H2630, RIEGL VZ-400, and GigaView monitoring units.

Monitoring Unit	Specifications
NEC-H2630	Measuring range (°C): −40 to 500
	Resolution (°C): 0.04 °C or better (at 30 °C, ∑16 *)
	Accuracy: ±2% of reading
	Spectral range (μm): 8 to 13
	Focusing range: 30 cm to infinity
	Thermal image pixels: 640 (H) × 480 (V)
RIEGL VZ-400	Maximum pulse repetition rate (PRR) (kHz): 300 (high-speed model)
	Effective measurement rate (meas./sec): 122,000 (high-speed model)
	Minimum range (m): 1.5
	Maximum range (m): 600 (long-range model)/350 (high-speed model)
	Accuracy | Precision (mm): 5/3
	Laser wavelength (nm): 1550
	Laser beam divergence (mrad): 0.3
GigaView	Resolution: 1280 × 1024
	Frame-rates (fps): 50 to 17,000
	Shutter: 1/50–1/100,000
	Sensor: 10-bit mono or 24-bit color

* Signal to noise (s/n) improvement by ensemble averaging of 16 images captured.

**Table 3:** Times series of $\bar{\Delta TIR}$ for region of interest (ROI) and room temperature.

Time (h)	$\bar{\Delta \mathrm{TIR}}$ (°C)	Room Temperature (°C)	Time (h)	$\bar{\Delta \mathrm{TIR}}$ (°C)	Room Temperature (°C)
0.0	0.000	23.3	5.3	0.578	23.3
1.2	0.002	23.3	5.4	0.638	23.3
1.5	0.002	23.3	5.7	0.653	24
1.8	0.032	23.3	6.0	0.649	24
2.0	0.025	23.3	5.3	0.578	24
2.3	0.073	23.3	6.3	0.755	24
2.8	0.131	23.3	6.5	0.875	24
3.0	0.145	23.3	6.8	1.119	23.5
3.3	0.229	23.3	7.0	1.197	23.5
3.5	0.291	23.3	7.3	1.179	23.5
3.8	0.319	23.3	7.5	1.145	23.5
4.0	0.331	23.3	7.8	1.135	23.5
4.3	0.435	23.3	8.1	1.106	23.5
4.5	0.466	23.3	8.3	1.045	23.5
4.8	0.480	23.3	8.7	1.098	23.5

Note: location of the ROI is shown in Figure 6.
